# Health improvements of type 2 diabetic patients through diet and diet plus fecal microbiota transplantation

**DOI:** 10.1038/s41598-022-05127-9

**Published:** 2022-01-21

**Authors:** Lili Su, Zhifan Hong, Tong Zhou, Yuanyuan Jian, Mei Xu, Xuanping Zhang, Xiaoyan Zhu, Jiayin Wang

**Affiliations:** 1 College of Electronics and Information Engineering, School of Computer Science and Technology, Xi’an Jiaotong University, Xi’an, Shaanxi 710048, People’s Republic of China; 2 Guangdong Quantum Hi-Tech Microecological Medical Co., Ltd, Guangzhou, Guangdong 510030 People’s Republic of China; 3 Yunnan Richland International Hospital, Kunming, Yunnan 650224 People’s Republic of China

**Keywords:** Computational biology and bioinformatics, Microbiology, Diseases, Endocrinology, Health care

## Abstract

Type 2 diabetes (T2D) is a major public health problem, and gut microbiota dysbiosis has been implicated in the emergence of T2D in humans. Dietary interventions can indirectly influence the health status of patients with type 2 diabetes through their modulatory effects on the intestinal microbiota. In recent years, fecal microbiota transplantation is becoming familiar as a new medical treatment that can rapidly improve intestinal health. We conducted a 90-day controlled open-label trial to evaluate the health improvement ability of a specially designed diet, and the diet combined with fecal microbiota transplantation (FMT). According to our study, both diet and diet plus FMT treatments showed great potential in controlling blood glucose and blood pressure levels. Sequencing the V4 region of 16S rRNA gene on the Illumina MiniSeq platform revealed a shift of intestinal microbial community in T2D patients, and the changes were also observed in response to the treatments. FMT changed the gut microbiota more quickly than diet. Beneficial bacterium, such as *Bifidobacterium*, increased along the study and was negatively correlated with blood glucose, blood pressure, blood lipid and BMI. Sulfate-reducing bacteria (SRB), *Bilophila* and *Desulfovibrio*, decreased significantly after treatment, showed a positive correlation with blood glucose indices. Thus, the specially designed diet is beneficial to improve blood glucose control in diabetic patients, it also showed the potential to reverse dyslipidemia and dysarteriotony.

## Introduction

Type 2 diabetes (T2D) is a form of diabetes generally characterized as elevated blood glucose levels, insulin resistance and relative lack of insulin. T2D is the most common type of diabetes, which is often diagnosed in older adults, and is increasingly seen in children, adolescents and younger adults due to rising levels of obesity, physical inactivity and poor diet. In fact, the incidence rate of T2D has increased markedly since 1960 in parallel with obesity^1^. It is generally known that a combination of genes and lifestyle results in T2D^2^. Recent insights provided the evidence of gut microbiota involvement in T2D, although findings diverge among studies^3–5^.

Diet plays an important role in shaping the intestinal microbiota and the gut responds very rapidly to alterations in diet^6,7^, thus diet may serve as a potential new target for disease control related to gut microbes^8–11^. In recent years, prebiotics, as a kind of dietary fiber, has received much attention. The relationships between prebiotics, probiotics and the gut microbiome have been deeply studied with the help of high-throughput sequencing techniques^12–14^. Dietary fibers may reduce the risk of developing type 2 diabetes and insulin resistance, which is most likely mediated by human gut microbiome^11,15,16^. Gut microbiota may regulate blood sugar levels through multiple mechanisms, including gut permeability and endotoxemia, production of short-chain fatty acids (SCFAs) and branched-chain amino acids (BCAAs), and perturbation of bile acid metabolism^17^.

Fecal microbiota transplantation (FMT) has now become widely accepted as a highly successful rescue treatment for *Clostridioides difficile* infection (CDI)^18^. FMT is also proved to be beneficial as a treatment for many other diseases, such as Ulcerative Colitis (UC)^19,20^, Irritable Bowel Syndrome (IBS)^21,22^, and other gastrointestinal disorders^23,24^. In addition, FMT from healthy donors into patients with metabolic syndrome also results in increased microbial diversity and improved glycemic control, as well as insulin sensitivity^25^. FMT as a supplement to dietary therapy was also being tried to treat diseases in recent years^26,27^. Whether FMT can enhance the beneficial effects of diet by altering gut microbiota of T2D patients has not been investigated.

In this study, we conducted an observational study to evaluate the safety and efficacy of a new diet consisting of probiotics, prebiotics and whole grains (the PPW formulation), and FMT combination treatment method of T2D. Furthermore, we then applied a microbiome-wide association study to characterize the changes in the microbial community response to the treatments, to reveal the relationship between treatments and microbiota alternation.

## Methods

### Study design and participants

The present study was designed as a 90-days controlled open-label trial, a random sample of patients with T2D was recruited from society, and a questionnaire was provided to determine whether the registered participants met the inclusion or exclusion criteria. Inclusion criteria were as follows: (1) Formal diagnosis with type 2 diabetes for at least 12 months before the start of the trial. (2) No history of smoking and alcohol abuse. (3) A minimum age of 18. And exclusion criteria were: (1) Antibiotic exposure in the previous 3 months. (2) Consumption of any probiotic or prebiotic products during the 3 months prior to commencement of the study. (3) Other gastrointestinal diseases were diagnosed. (4) Severe mental-health problems prior to enrolment. (5) Severe organic diseases. (6) Infectious diseases. (7) Pregnant women. Matters needing attention and possible related risks of this study were given in writing. The present study was conducted according to the guidelines laid down in the Declaration of Helsinki, and all procedures involving human participants were approved by the Ethics Committee for the Yunnan Richland international hospital (Ethical Approval Number: 2017-008). Written informed consent was obtained from all subjects before being enrolled in the study. This trial was registered on the Chinese Clinical Trail Registry, with the registration number ChiCTR2100051257 on 17/09/2021.

A total of 25 patients responded to the recruitment at Yunnan Richland international hospital from October 2017 to March 2018, five individuals were excluded from the study on the basis of criteria. Finally, 16 diabetic patients were enrolled in the study. All the participants were aged between 41 and 76 at the beginning of the study. At the start of the experiment, participants were randomly assigned 1:1 to the diet-only (group D) or diet-FMT group (group DF) using an online randomization tool^28^. Participants in group D received the PPW formulation orally three times a day as part of their normal diet. The first 20 days were the observation period of intensive intervention, all participants were concentrated in the hospital for intervention in this time. The latter 70 days are the home intervention period, during this time, the participants did not strictly follow the dietary intervention. Day 0 is the baseline of the intervention, day 20 is the end of the strict intervention time point and day 90 is the end of the whole experiment.

**Figure 1:**
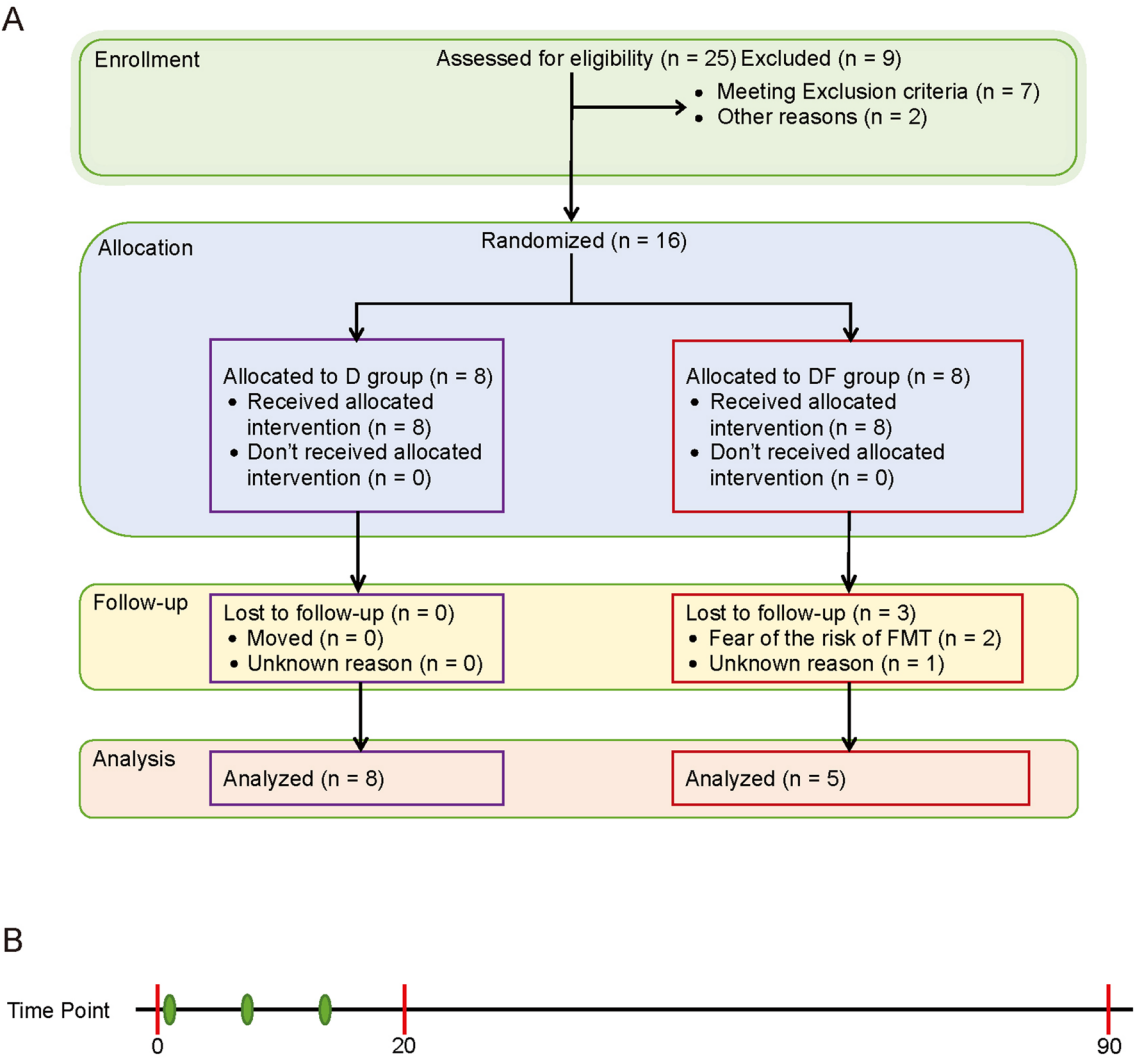
Trail profile. (**A**) Participant flow diagram of the study. (**B**) Time points of the current study, red points represent the time points of sample collection, green points represent the time points of FMT.

Participants in group DF were provided the same diet as group D coupled with fecal microbiota transplantation. Patients received FMT for once a week along the first 3 weeks of the intervention. Unfortunately, 3 participants from group DF dropped out of the trial because of the time commitment. Finally, 13 participants complete the whole study (Fig. 1, Supplementary Table S2).

### Dietary formulations

The compound microorganism preparation PPW consisted of probiotics, prebiotics and whole grains, including three ready-to-consume prepared foods (Liangzitianjian formula No. 1, No. 2 and No. 3; Guangdong Quantum Hi-Tech Microecological Medical Co., Ltd, China). Concretely, formula No. 1 is a combined dietary fiber powder preparation (15 g per bag) containing resistant dextrin, inulin, galactooligosaccharide (GOS), fructo-oligosaccharide (FOS) and xylooligosaccharide. Formula No. 2 is pre-processed whole grain powder mixture (25 g per bag) containing organic wheat, oat and highland barley. Formula No. 3 is synbiotics powder preparation (4 g per bag) containing resistant dextrin, GOS, stachyose, fermented fruit and vegetable freeze-dried powder, *Lactobacillus acidophilus NCFM*, *Bifidobacterium lactis HN019* and *Lactobacillus paracasei Lpc-37*. The total amount of probiotics is 1.00E + 10 per bag. The detailed components were listed in Table S1. All components are approved as food ingredients in China. All participants kept their original medication and the Insulin dependent patients adjusted the doses according to the guidance of the clinician. Before the first FMT, the participants accepted formula No. 1 (8 bags, 120 g) for bowel cleansing.

### Fecal microbiota transplantation

FMT was prepared from stools donated by four healthy volunteers. The volunteers were between 18 and 30 years of age, negative T2D, and otherwise healthy, as assessed by a screening questionnaire. Donor stools were screened for enteric pathogens, parasites, and *C. difficile* toxin. The donors were negative for HIV, hepatitis A IgM, hepatitis C antibody, hepatitis E IgM, cytomegalovirus antibody, and syphilis. All donors were prospectively screened and rescreened every 3 months. Donors who received antibiotics within 3 months of stool collection were excluded. Microbiota isolation was performed within 1 h of stool collection using the Fecal analysis pre-treater TG-01 (Guangzhou Treatgut Biotechnology Co., Ltd, China). About 30 g of isolated bacterial precipitation was collected.

The transplants were performed at day 1, 8, and 15 respectively after the start of the trial. All participants were asked to swallow 30 capsules on an empty stomach under medical supervision, each capsule contains approximately 1 g of the isolated bacterial precipitation. Capsules were thawed in a water bath at 37 °C for 10 min before swallowing. It took about 90 min to swallow all 30 capsules. No adverse reactions such as abdominal pain and diarrhea were reported during this study.

### Sample collection

Longitudinal samples were collected from each subject at an interval of 3 months. Stool and blood samples were collected at day 0, 20 and 90. Day 0 samples were collected one day before the trial as the baseline. Stool was passed in to a paper box collection container covering the bowl of the toilet. Wearing gloves, participants scooped the stool into empty sterile feces collection containers. Stool samples were immediately frozen and stored at − 80 °C freezer prior to DNA extraction. The blood samples were drawn using standard venipuncture techniques by experienced phlebotomists from the Yunnan Richland hospital. After collection, blood samples were left at room temperature for 20 min to allow the blood to clot. The samples were then centrifuged (2000×*g* for 10 min). Serum was aliquoted into tubes and were tested with biochemistry techniques. Participants weighted themselves every morning on an empty stomach.

### DNA extraction and 16S ribosomal DNA sequencing

Total DNA was extracted from 0.25 g of fecal samples using a QIAamp Fast DNA Stool Mini Kit (QIAGEN) according to the manufacturer’s instructions. For each sample, the V4 region of the 16S rRNA gene was amplified by PCR using primers 515F: (5′-GTGYCAGCMGCCGCGGTAA-3′) and 806R: (5′-GGACTACNVGGGTWTCTAAT-3′), PCR reactions were performed using KAPA HiFi HotStart DNA Polymerase. Thermal cycle consisted of an initial denaturation at 95 °C for 3 min, thirty cycles of denaturation at 95 °C for 20 s, annealing at 60 °C for 30 s, extension at 72 °C for 30 s and a final extension step at 72 °C for 5 min. Amplicons of about 290 bp were purified with a magnetic bead-based clean-up system (VAHTS DNA Clean Beads; Vazyme) and sequenced on Illumina MiniSeq platform using a 2 × 150 bp paired end protocol, according to the manufacturer’s instructions (Illumina).

### Bioinformatics and statistics

Paired-end reads were merged using FLASH (v1.2.11)^29^. After merged, base sequence quality information was confirmed by FastX Toolkit (http://hannonlab.cshl.edu/fastx_toolkit/index.html) and reads with base quality scores below the minimum (20 per base) score across the whole read or read length less than 200 bases were removed. A total of 2,227,090 high-quality filtered reads were obtained, with a mean of 57,105 reads per sample. For each sample, 30,000 high-quality filtered reads were selected randomly for subsequent analysis. Briefly, filtered reads (Table S3) were binned into operational taxonomic units (OTUs) at a sequence similarity level of 97% by UCLUST^30^, OTUs less than 2 reads mapped were removed. Taxonomy was assigned using the RDP (Ribosomal Database Project) classifier against Greengenes database (May 2013 release). Chimera filtering was performed through VSEARCH^31^ by discarding all singleton OTUs, alpha (Supplementary Table S4) and beta diversity were analyzed through the QIIME2 pipeline (version 2018.8.0)^32^.

All statistical analyses were performed in R (version 3.4.3) or STAMP^33^. Following the intervention, differences across treatment groups were assessed using independent-samples *t* test for the comparisons of BMI (at baseline), gender, and baseline outcome measurement. Changes in measured variables from baseline to the end of the study within each group were also assessed using paired-samples *t* test. To compare microbial beta diversity between samples, Weighted Unifrac distance matrices were measured using QIIME2. Principal coordinate analysis (PCoA) was applied on the resulting distance matrices to generate two-dimensional plots using STAMP. Significant differences in beta diversity were assessed using two tailed Wilcox matched-pair signed-rank test. Changes in Stool bacterial OTU abundances were determined using Kruskal–Wallis *H* test (Multiple groups), followed by the Tukey–Kramer post hoc test without correction. p < 0.05 was considered statistically significant. Spearman correlation analysis was performed to analyze the correlation between the gut microbiota and clinical characters.

## Results

### The PPW formulation were beneficial to weight, blood glucose and blood pressure in T2D patients

Between October 2017 and March 2018, we recruited 25 individuals who were diagnosed with type 2 diabetes for at least 12 months before the start of the trail. 7 individuals were excluded from the study according to the exclusion criteria we set and 2 individuals quit the trail. As a result, 16 individuals were enrolled in our study. At the beginning of the trail, 3 participants in DF group were lost to follow up (Fig. 1). Finally, we collected 13 participants’ biochemical indices and fecal microbiome data. Biochemical indices of day 90 were unavailable for one participant in D group.

In order to explore whether the dietary intervention can improve the health status of T2D patients, biochemical parameters of 13 T2D patients were measured at the Yunnan Richland hospital (Supplementary Table S5). Serum glucose, serum C-peptide, triglyceride, total cholesterol, high density lipoprotein and low-density lipoprotein were measured using an automatic biochemical analyzer (AU480 Clinical Chemistry System, Beckman Coulter, CA, USA) and HbA1c levels were measured by high-performance liquid chromatography (Bio-Rad Variant II Turbo, Bio-Rad Laboratories, Munchen, Germany). There were no significant differences among treatment groups at baseline except for total cholesterol (Table 1). Total cholesterol of Participants in the DF group was significantly higher than the D group. We are uncertain the cause of this significant differences as to subjects were randomized into treatment groups.

**Table 1:** Baseline characteristics of subjects that completed the study (N = 13).

Baseline data	Treatment group		p-value
	D	DF	
Subjects (N)	8	5	
Gender (M/F)	6/2	3/2	0.63
Age (years)	60.4 ± 12.0	57 ± 13.2	0.65
BMI (kg/m^2^)	24.8 ± 3.0	25.2 ± 5.0	0.89
Fasting blood glucose (mmol/L)	9.6 ± 3.8	7.1 ± 1.3	0.12
2-h postprandial blood glucose (mmol/L)	15.4 ± 4.6	12.0 ± 2.7	0.12
Glycosylated hemoglobin (%)	8.3 ± 1.7	6.9 ± 1.1	0.11
Total cholesterol (mmol/L)	4.72 ± 0.97	4.93 ± 1.18	0.0018
Triglyceride (mmol/L)	1.57 ± 0.83	1.96 ± 0.94	0.46
High-density lipoprotein (mmol/L)	1.41 ± 0.27	1.20 ± 0.33	0.27
Low density lipoprotein (mmol/L)	2.66 ± 0.94	3.18 ± 0.86	0.33
Systolic blood pressure (mmHg)	135 ± 14	133 ± 16	0.84
Diastolic blood pressure (mmHg)	79 ± 11	78 ± 10	0.91

Values are expressed as means ± standard deviation. *D* diet-only, *DF* diet-FMT.

Two-tailed Student’s *t* test for independent-samples were used to compare anthropometric values and biochemical values between groups.

**Figure 2:**
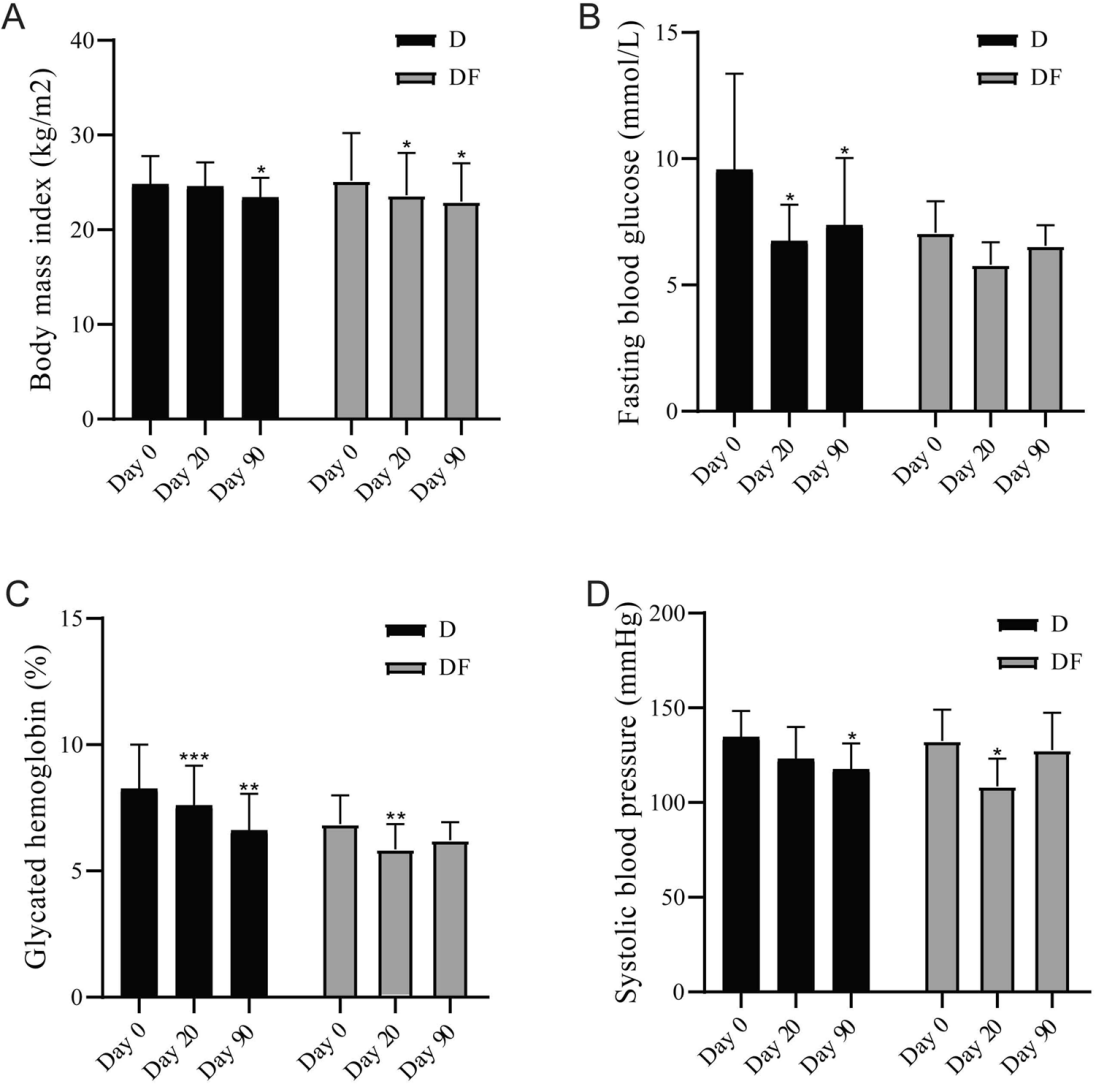
Body measurement and biochemical indices changed by treatments. (**A**) The levels of body mass index at different time points in each group. (**B**) The levels of fasting blood glucose at different time points in each group. (**C**) The levels of glycated hemoglobin at different time points in each group. (**D**) The levels of systolic blood pressure at different time points in each group. *p < 0.05 vs baseline; **p < 0.01 vs baseline; ***p < 0.001 vs baseline. Two-tailed Student’s *t* test for paired-samples.

After 90 days of treatment, PPW significantly decreased in body mass index (BMI) from baseline in participants of group D and DF (23.4 ± 2.1 vs 24.8 ± 3.0 and 23.0 ± 4.1 vs 25.2 ± 5.0 kg/m^2^; p < 0.05 for D and DF vs baseline). An earlier effect of weight loss was shown in participants in group DF, which showed weight loss at 20 days of intervention (23.6 ± 4.5 kg/m^2^; p < 0.05 for DF vs baseline) (Fig. 2A). Compared to group D, which showed weight loss effect after 90-day intervention. PPW diet significantly improved glycemic control in T2D patients, the diet intervention significantly lowered the fasting blood glucose (FBG) and glycosylated hemoglobin (HbA1c) of participants in group D. FBG fell sharply during the first 20 days (6.7 ± 1.4 vs 9.6 ± 3.8 mmol/L; p < 0.05 for day 20 vs baseline), the strict dietary management phase. Followed by small increases during the self-control phase (7.4 ± 2.7 mmol/L for day 90) (Fig. 2B). HbA1c kept falling on day 20 and day 90 in participants of D group (7.6 ± 1.6 and 6.6 ± 1.5 vs 8.3 ± 1.7%; p < 0.001 for day 20 vs baseline and p < 0.01 for day 90 vs baseline). While in DF group, HbA1c decreased at day 20 (5.9 ± 1.0 vs 6.9 ± 1.1%; p < 0.01 for day 20 vs baseline), and then followed by small rebound at day 90 (6.2 ± 0.7%) (Fig. 2C). Systolic blood pressure decreased in both groups (118 ± 14 vs 135 ± 14 mmHg, p < 0.05 for day 90 vs baseline in D group; 109 ± 15 vs 133 ± 16 mmHg, p < 0.01 for day 20 vs baseline in DF group) (Fig. 2D). p < 0.05 was considered statistically significant. Blood lipid levels, both total cholesterol and triglyceride, displayed decreasing trends during intervention. Total cholesterol decreased significantly in D group at day 20, other comparisons with baseline did not differ significantly both group (Supplementary Fig. 1).

To further explore the role of fecal microbiota transplantation played in our observational study, we also compared the effects of two kinds of intervention between DF group and D group. Although paired *t* test showed statistic differences at different time points (day 20 and day 90 when compared with baseline) between DF and D groups, comparing the biochemical indices of participants in DF and D groups in adjusted mean changes from baseline at the same period, no significant differences were shown. That's probably because of the small sample size.

### Overall gut microbiota response to diet and FMT

**Figure 3:**
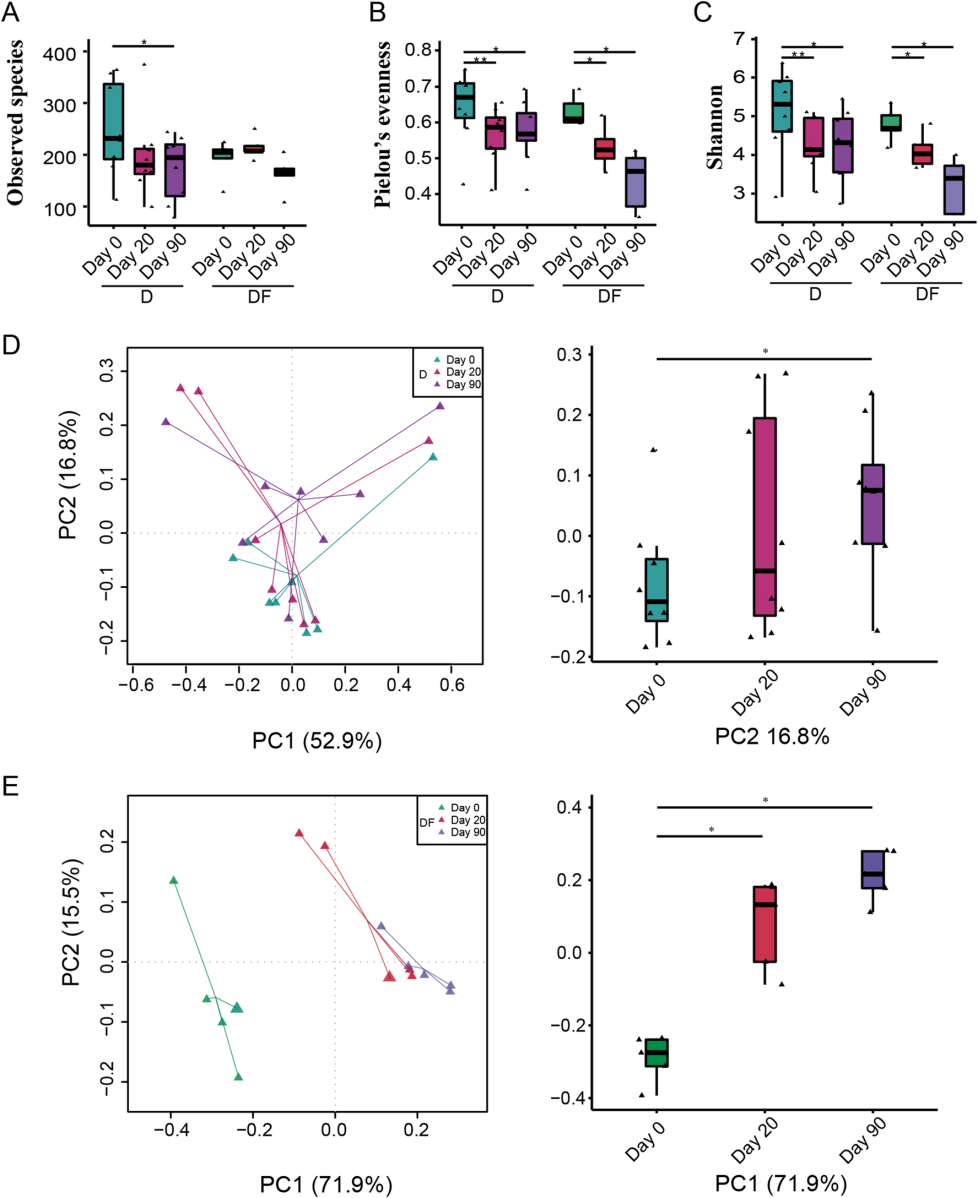
Gut microbiota diversity. Alpha diversity quantified by the (**A**) Observed species index, (**B**) Pielou’s evenness index, (**C**) Shannon index after rarefying to 3000 sequences. (**D**) Weighted Unifrac PCoA of gut microbiota based on the OTU data in D group. (**E**) Weighted Unifrac PCoA of gut microbiota based on the OTU data in DF group. *p < 0.05; **p < 0.01. Two-tailed Student’s *t* test for paired-samples was used in alpha diversity comparison. Two tailed Wilcox matched-pair signed-rank test was used in beta diversity comparison.

To investigate changes in the gut microbiome during the intervention, we performed 16S V4 rRNA gene sequencing on 39 fecal samples from T2D patients. A total of 2,227,090 high-quality reads were obtained, with a mean of 57,105 reads per sample. After merged, 30,000 high quality reads were used for subsequent analysis. Reads were clustered in OTUs at 97% identity. Gut microbiome was characterised by a significant reduction of alpha diversity in both groups. There was no significant change in the observed species richness, except day 90 vs day 0 in D group (p < 0.05), indicating that species richness was not much affected by the intervention (Fig. 3A). However, species evenness was decreased in both groups (Fig. 3B), suggesting that the distribution of gut microbial species has changed dramatically. As a result, the Shannon index, who reflecting the overall diversity of species, was decreased as the same trend with Pielou’s evenness (Fig. 3C). Compared to dietary interventions alone, FMT plus dietary interventions had a smaller effect on the reduction of diversity.

Principal coordinates analysis (PCoA) based on weighted UniFrac distances showed a significant segregation among samples from 3 time points, especially in DF group, confirming the presence of compositional differences in the gut microbial structure of participants before and after intervention. The gut microbiome of patients at day 20 became separate to the baseline immediately following treatment, and continued to be affected by the intervention on day 90. Particularly, it showed significant differences between day 0 and day 20 in DF group, the difference was reflected in PC1 (Wilcoxon matched-pair signed-rank tests (two tailed), p < 0.05), which accounts for 71.9% of the total variability (Fig. 3E). However, in D group, samples of day 20 compared to day 0, the difference was not observed. These results indicated that dietary intervention can slowly alter the structure of gut microbiome, while the effect of FMT on microbiota change was relatively rapid. After a longer duration of intervention, at day 90, intestinal microbial structure of participants from D group and DF group showed differences from the baseline to a different extent (Fig. 3D,E).

### Fecal microbiota changes upon PPW diet and FMT

**Figure 4:**
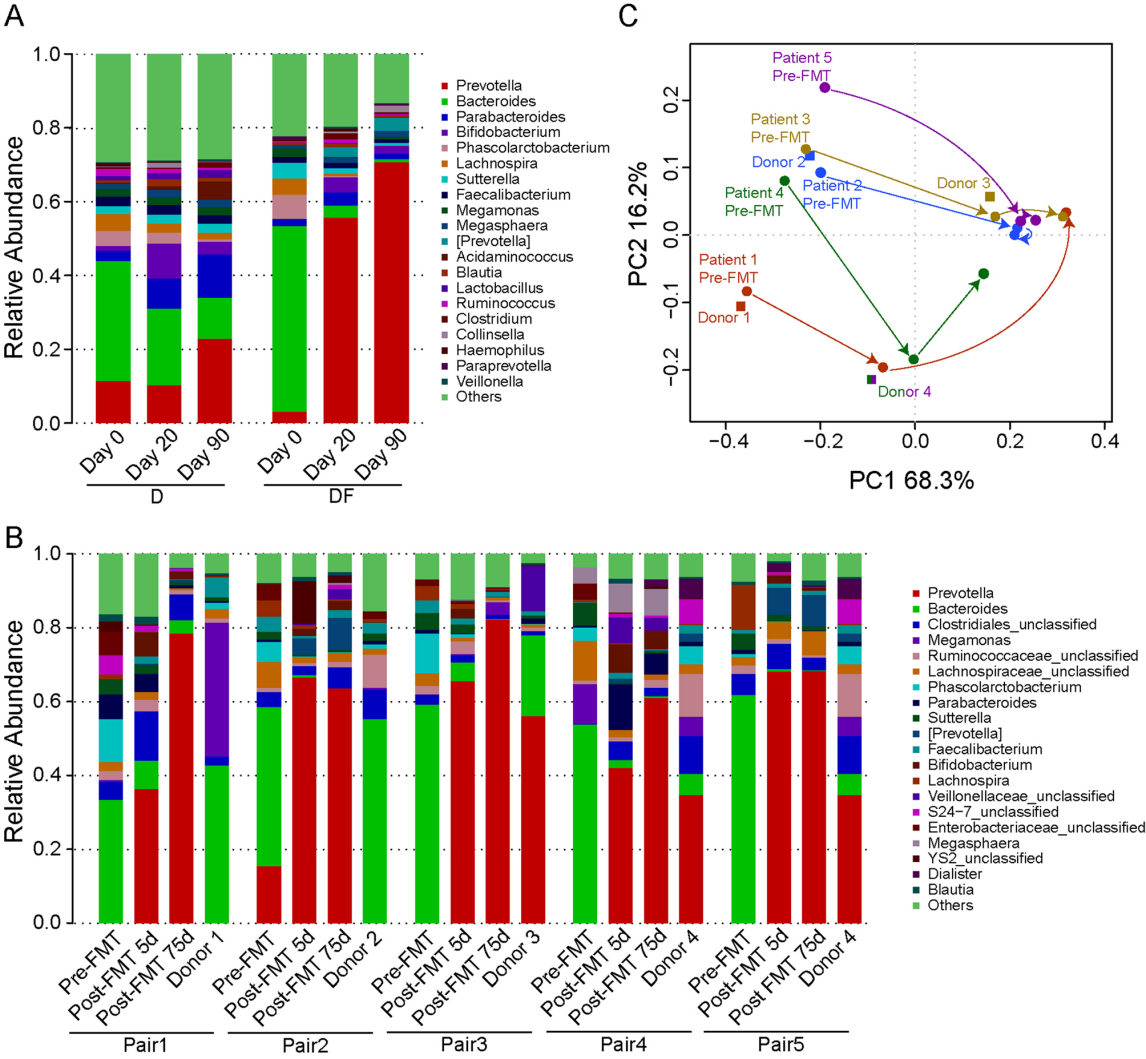
Changes of the composition of fecal microbiota. (**A**) Bacterial composition at the genus level in each group. (**B**) Bacterial composition at the genus level of each person receiving the FMT and the paired donor. (**C**) PCoA of weighted UniFrac distances before and after FMT.

To determine whether this alteration of gut microbiome will affect intestinal health, we explored the abundances of gut community composition in different periods at the genus level. *Bacteroides* was the most abundant genera in D (32.5%) and DF (50.4%) group samples at day 0, and it has maintained its dominant position in D group until day 20. However, in DF group, *Prevotella* replaced *Bacteroides* as the dominant genera at day 20. At the end of the trail, *Prevotella* became the most abundant genera in both group (22.9% in D group and 70.8% in DF group) (Fig. 4A). To quantify and compare community similarity and taxonomic character of the gut microbiome at different time points, nonparametric Kruskal–Wallis with Tukey–Kramer post-hoc test was performed using STAMP, p < 0.05 was considered statistically significant. The D group yielded significantly increased relative abundances of *Acidaminococcus*, *Bifidobacterium*, *Blautia* and *Pseudomonas*, while the abundances of *Bilophila*, *Oscillospira*, *Roseburia* and *Ruminococcus* were significantly decreased (Supplementary Table S6). Compared with D group, the abundances of bacteria in DF group altered even more, *Bifidobacterium*, *Collinsella*, *Lactobacillus* and *Prevotella* significantly increased, *Bacteroides*, *Bilophila*, *Lachnospira*, *Odoribacter*, *Phascolarctobacterium* and *Sutterella* decreased (Supplementary Table S7), they were affected greatly by the dramatic abundance change of *Bacteroides* and *Prevotella*. It was noteworthy that the abundance of *Bifidobacterium* increased significantly and *Bilophila* decreased significantly in both groups during the intervention.

Importantly, stool samples were collected from the healthy FMT donors (Supplementary Table S8) to assess the composition of the microbiome before and after intervention via 16S ribosomal RNA gene sequencing. The microbiota of recipient patients in this study was characterized by the high abundant of *Prevotella* at day 20, no matter which genera was the dominant bacterium in their donors. In all the pairs of FMTs, 3 out of 5 donors’ intestinal microbiota was dominated by *Prevotella* (Fig. 4B). Principal coordinates analyses (PCoA) of weighted UniFrac distances demonstrated that the gut microbiome of 3 patients (Patient 3, 4, 5) became most similar to the FMT donor immediately following treatment, but in both cases later deviated away though remaining distinct compared to pre-FMT. What all three cases had in common was that their donors were dominated by *Prevotella*. The remaining 2 cases (patient 1, 2) appeared to be away from the donor and keep moving away until the end of trail. The cases had a Bacteroides dominated gut microbiota (Fig. 4C).

### The gut microbiome of T2D patients was associated with clinical indices

**Figure 5:**
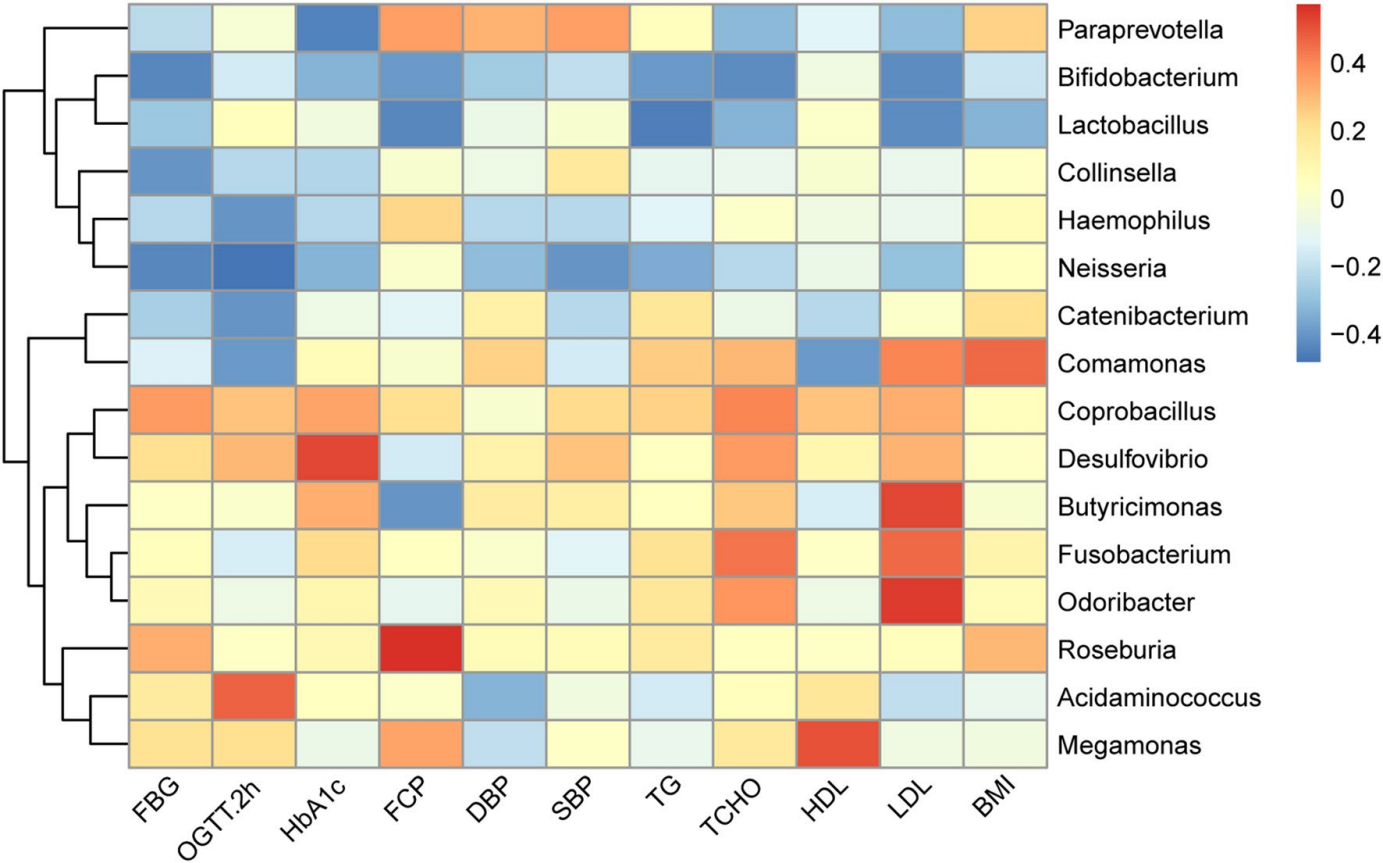
Heat map of identified key genus responding to the treatments and Spearman’s correlation between identified genus and biochemical indices. The color of the spots represents R-value of Spearman’s correlation between the genus and biochemical indices.

In order to identify interactions between blood biochemical indices, physical measurement indices and the microbiota, we first conducted a Spearman correlation coefficient test to assess the possible relationship between these indices and microbial profiles. The genera with a correlation coefficient greater than 0.4 or less than -0.4 with the indices were shown in Fig. 5. The strongest correlations between clinical outcomes and bacteria were observed for *Bifidobacterium*, which showed negative correlations with FBG, OGTT.2h, HbA1c, FCP, DBP, SBP, TG, TCHO, LDL and BMI. *Bifidobacterium* appeared to confer a health benefit to T2D patients. Likewise, *Lactobacillus* also showed negative correlations with FBG, blood lipid and BMI. In addition, *Collinsella* and *Neisseria* showed negative correlations with blood glucose levels as well. *Desulfovibrio* was positively related to blood glucose indices. *Butyricimonas*, *Fusobacterium* and *Odoribacter* correlated positively with changes in blood lipid indices, especially the low density lipoprotein.

**Figure 6:**
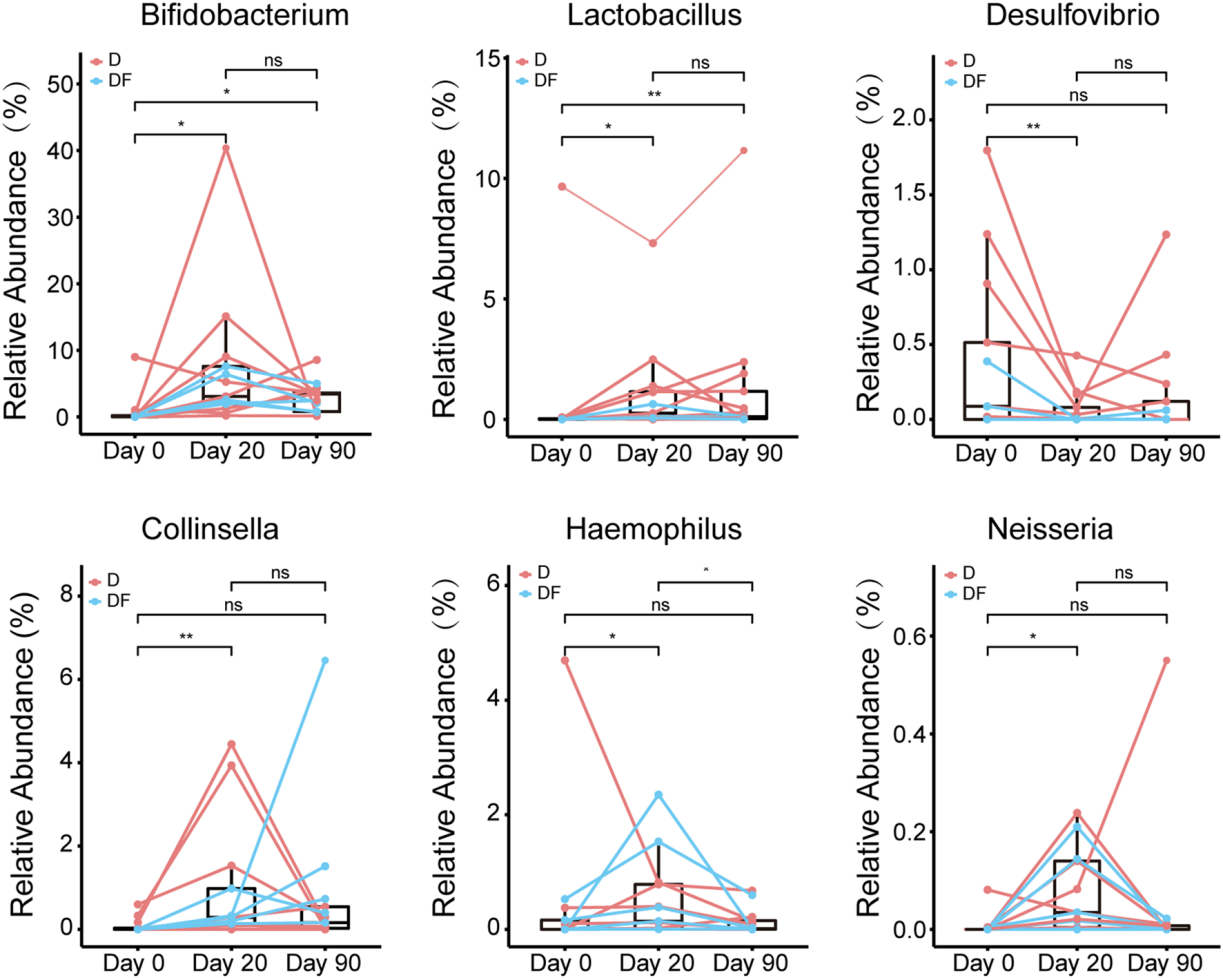
Metagenomic biomarkers associated with diet and diet plus FMT intervention. The relative abundance of selected genus were plotted for each subgroup. *p < 0.05; **p < 0.01. Two tailed Wilcox matched-pair signed-rank test was used for the statistical calculation.

Moreover, we observed that the relative abundances of bacteria closely related to clinical outcomes changed significantly before and after intervention. It was noteworthy that both *Bifidobacterium* and *Lactobacillus* increased significantly, and the change continued through the end of the intervention. By comparing the changes of the relative abundance of *Collinsella*, *Haemophilus* and *Neisseria* during intervention, we found that compared with day 0, in the middle intervention, these bacteria significantly increased. Conversely, *Desulfovibrio* was significantly decreased by the PPW diet and FMT treatment (Fig. 6).

## Discussion

In the current study, we explored the alterations of 13 T2D patients’ physical conditions with two intervention strategies, combining with the changes of gut microbiome. In previous study, diet was often used as an intervention for the gut microbiota, and duration of intervention was usually only 1–6 weeks, the long-term effects were less mentioned^9,34–36^. As a new therapeutic method targeting intestinal microbiota, in recent years, FMT has been used to try to treat an increasing number of diseases. The health effects of an FMT combined diet have not been fully studied. Given this, we carried out a 90 day’s PPW dietary intervention, to deep explore the effects of long-term dietary intervention on the health of T2D patients. Furthermore, we overlayed the diet with FMT treatment, to find out if FMT is helpful to diet treatment.

As expected, in this 90-day study, both diet-only and FMT combination treatments were associated with significant weight loss. Some beneficial effects on blood glucose control were also observed, both treatments were associated with reductions in fasting blood glucose and glycated hemoglobin. Although the mean change of fasting blood glucose was slightly but not significantly greater in the DF group, glycated hemoglobin changed significantly in both groups. This finding was consistent with previous studies, the effect of the intervention diets on glycemia appears to be related to weight reduction^37–39^. This reflects the glycemic control ability of PPW diet on T2D patients, making it an alternative strategy for diabetes management. In addition, significantly reductions were seen in systolic blood pressure value in both groups. This may be related to the higher intake of dietary fiber, earlier studies pointed out that dietary fiber intake was inversely related to blood pressure^40,41^. Plasma lipid parameters, including total cholesterol and triglyceride, also showed a certain control trend with dietary intervention, which had been significantly ameliorated by probiotic in the other study^42^. Thus, the PPW diet seems also likely to induce cardiovascular effects.

Although both groups had the same effect, the onset time of the two groups was different. Under the action of FMT, DF group showed a faster effect on weight loss. Most previous studies have shown that body weight was closely related to gut microbiota^43–46^. This might be the result of supplementation of the diet with probiotic organisms and prebiotic compounds that influence bacterial growth. FMT might also contribute to the weight loss by introducing gut microbiota from healthy individuals into the gut of the patients. Previous study has confirmed that autologous FMT maintains weight loss with specific dietary interventions^26^. No significant differences of the biochemical indices of participants were found between D and DF groups when comparing the adjusted mean changes from baseline at the same time. The small sample size may be the reason for this result. Comparing the data at different period in this study, we found that the health improvement effects were trend to rebound at the end of the intervention. It suggested that the improvement of clinical indicators observed after short-term intervention was transient, long-term regulation was needed to maintain the effect of blood glucose control.

Concomitant with the improved glucose homeostasis, we observed altered microbial composition induced by the intervention. It is generally considered that greater overall diversity implies better health^47^, however, consistent with Zhao et al.^11^, our finding challenged the current notion. In our study, the overall diversity of gut microbiota was decreased. Feng et al. noted that colorectal cancer patients had a higher alpha diversity of intestinal microbiome than healthy populations, indicating that the presence of inflammation would either suppress the growth of beneficial bacteria or promote the growth of those that are deleterious^48^. Consumption of probiotics *VSL#3* decreased TNBS-induced colitis but reducing the gut microbial diversity in mice^49^. Perhaps we should be skeptical of the conclusion that a less diverse microbiota is less "healthier" for the host. Meanwhile, we noted that FMT mitigated the effects of dietary intervention on reducing the alpha diversity of gut microbiome. Mocanu’s study also showed that fiber intake alone reduced intestinal microbial diversity, while fiber supplementation after FMT could mitigate the reduction in diversity or even increase it^50^. We found that compared to the diet, FMT changed the structure of intestinal microbiota faster. This is also consistent with the findings of Mocanu’s study. Former studies have shown that the long-term intake of fat-rich diet was associated with increases in Bacteroides and the vegetarian was beneficial to the proliferation of *Prevotella*^51^. *Prevotella* were also found to be associated with dietary fiber-induced improvement in glucose tolerance^52^, which was consistent with our findings. During the 90-day intervention study, the gut microbiome of T2D patients changed from *Bacteroides* predominates to *Prevotella* predominates, this might be a positive and optimistic sign. With the reinforcement of FMT, *Prevotella* became the dominant species much faster.

Furthermore, in our study, the abundance of *Bifidobacterium* increased significantly in both D and DF groups during the intervention. *Bifidobacterium* may exert a range of beneficial health effects, including regulation of intestinal microbial homeostasis, modulation of local and systemic immune responses, inhibition of pathogens and harmful bacteria that colonize or infect the gut mucosa^53^, and improve the gut mucosal barrier and lower levels of lipopolysaccharide in the intestine^54^. *Bifidobacterium* also appears to be the most consistently supported by the literature genus containing microbes potentially protective against T2D^9,55^. In our study, the relative abundance of *Bifidobacterium* was negatively correlated with most clinical characters, including blood glucose, blood pressure, blood lipid and BMI, suggesting that *Bifidobacterium* might be a pivotal organism associated with the improvement of T2D. *Lactobacillus*, a common beneficial bacterium, was negatively correlated with FBG, blood lipid and BMI. In other reports, meta-analysis of RCT studies found that probiotic *Lactobacillus* improved weight management outcomes in obese adults^56^. Consumption of yogurt and other dairy products fermented by *Lactobacillus* was also significantly associated with protection from T2D and obesity^57^. As a typical lactic acid bacterium (LAB), *Lactobacillus*’s main fermentation end-product is lactate, which can ensure a control over less friendly bacteria by causing acidification in the gut^58^. The PPW diet contained *Lactobacillus* spp. and *Bifidobacterium* spp., which might contribute to the increased abundance of these bacteria in the intestine. Meanwhile, an increased fiber intake could also induce a higher abundance of *Bifidobacterium* and *Lactobacillus*, together with higher fecal butyrate levels^59^. *Bilophila* is a kind of sulfate-reducing bacteria (SRB), which are pro-inflammatory bacteria and have been shown to be involved in a number of inflammatory or immune diseases, including T2D^3^, metabolic syndrome^60^, and inflammatory bowel disease (IBD)^61^. The reduction of *Bilophila* decreased in the treatment groups predicted lower levels of inflammation. Another SRB, *Desulfovibrio*, showed a positive correlation with blood glucose indices, was also significantly decreased by the PPW diet and FMT treatment. This further demonstrates the improvement of intestinal microbiota in the intervention groups in our study. These results suggest that the enrichment of beneficial bacteria and reduction of pathogen-like bacteria might be involved in the amelioration of body health by PPW diet and FMT.

In conclusion, The PPW diet showed a potential benefit on reducing the body weight, fasting blood glucose and glycosylated hemoglobin in type 2 diabetic patients. In addition to blood sugar control, PPW diet also played roles in blood pressure and blood lipid regulation. FMT worked in conjunction with dietary intervention accelerated the weight loss effect, this may because of a faster intestinal microbiota change by FMT. It should be noted that in order to maintain the health benefits of the intervention, a long-term and radical dietary change is necessary. Alterations in the structure of the intestinal microbiome were involved in the health improvement. The main gut bacterium of T2D patients changed from *Bacteroides* to *Prevotella* after treatment. Beneficial organisms (such as *Bifidobacterium*) were significantly increased and harmful organisms (such as *Bilophila*) were significantly decreased. Our study provides a new potential therapeutic strategy for type 2 diabetes. Furthermore, hyperglycemia and hyperlipidemia may also be ameliorated by the treatment.

## Supplementary Information

Supplementary Figure 1.

Supplementary Tables.

## Data Availability

All raw sequences from this study were deposited in the National Center for Biotechnology Information (NCBI) Sequence Read Archive (SRA) under accession number PRJNA588354.
