# Supplementary material for: Health improvements of type 2 diabetic patients through diet and diet plus fecal microbiota transplantation
Source: Sci Rep. 2022 Jan 21;12:1152. doi: 10.1038/s41598-022-05127-9 (PMC8782834; doi:10.1038/s41598-022-05127-9)
Supplement: Supplementary file 1 — Supplementary Figure 1. [file 41598_2022_5127_MOESM1_ESM.pdf]

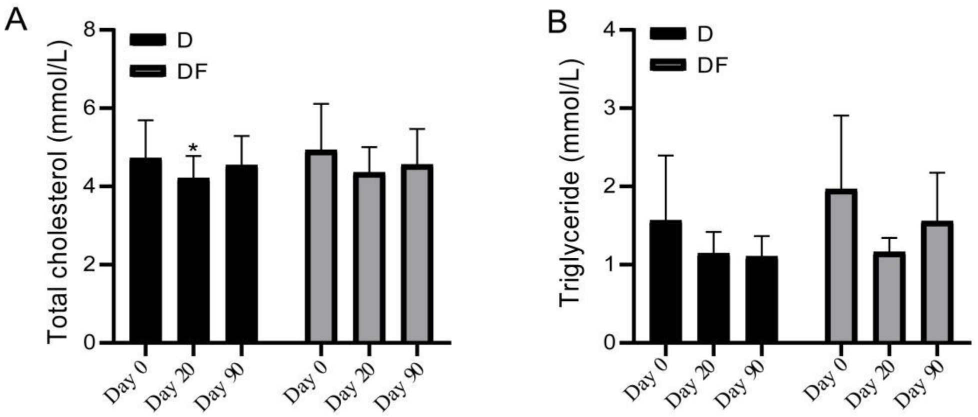

Supplementary Figure 1. Biochemical indices changed by treatments. (A) The levels of total cholesterol at different time points in each group. (B) The levels of triglyceride at different time points in each group. *p<0.05 vs baseline. Two-tailed Student’s t-test for paired-samples.
